# African Ancestry Gradient Is Associated with Lower Systemic F_2_-Isoprostane Levels

**DOI:** 10.1155/2017/8319176

**Published:** 2017-01-31

**Authors:** Francis Annor, Michael Goodman, Bharat Thyagarajan, Ike Okosun, Ayo Doumatey, Barbara A. Gower, Dora Il'yasova

**Affiliations:** ^1^Division of Epidemiology and Biostatistics, School of Public Health, Georgia State University, One Park Place, Suite 630, Atlanta, GA 30303, USA; ^2^Department of Epidemiology, Rollins School of Public Health, Emory University, 1518 Clifton Road, Atlanta, GA 30322, USA; ^3^Department of Laboratory Medicine and Pathology, University of Minnesota, 420 Delaware St SE b435, Minneapolis, MN 55455, USA; ^4^Center for Research on Genomics and Global Health, National Human Genome Research Institute, National Institutes of Health, Building 12A, Room 4047, 12 South Dr, MSC 5635, Bethesda, MD 20892, USA; ^5^Department of Nutrition Sciences, University of Alabama at Birmingham, 1675 University Blvd., Birmingham, AL 35294, USA

## Abstract

*Context*. Low levels of systemic F_2_-isoprostanes (F_2_-IsoP) increase the risk of diabetes and weight gain and were found in African Americans. Low F_2_-IsoPs could reflect an unfavorable metabolic characteristic, namely, slow mitochondrial metabolism in individuals with African ancestry.* Objective*. To examine differences in plasma F_2_-IsoPs in three groups with a priori different proportion of African ancestry: non-Hispanic Whites (NHWs), US-born African Americans (AAs), and West African immigrants (WAI).* Design*. Cross-sectional study.* Setting*. Georgia residents recruited from church communities.* Participants*. 218 males and females 25–74 years of age, who are self-identified as NHW (*n* = 83), AA (*n* = 56), or WAI (*n* = 79).* Main Outcome Measure(s)*. Plasma F_2_-IsoPs quantified by gas chromatography-mass spectrometry.* Results*. After adjustment for age, gender, obesity, and other comorbidities, WAI had lower levels of plasma F_2_-IsoP than AA (beta-coefficient = −9.8, *p* < 0.001) and AA had lower levels than NHW (beta-coefficient = −30.3, *p* < 0.001). Similarly, among healthy nonobese participants, F_2_-IsoP levels were lowest among WAI, followed by AA, and the highest levels were among NHW.* Conclusion*. Plasma F_2_-IsoPs are inversely associated with African ancestry gradient. Additional studies are required to test whether optimization of systemic F_2_-IsoP levels can serve as means to improve race-specific lifestyle and pharmacological intervention targeted to obesity prevention and treatment.

## 1. Introduction

Metabolic predisposition to obesity and type 2 diabetes among African Americans is well-established [1–3]. Identification of modifiable factors that predispose African Americans to these metabolic disorders may offer opportunities for targeted intervention. The importance of targeting race-specific metabolic predisposition is emphasized by the findings from weight loss interventions, showing that with similar treatments whether lifestyle-focused [2, 4, 5] or surgical [6, 7], African Americans tend to lose less weight than European Americans.

Our previous work examined the relationships between urinary F_2_-isoprostanes** (**F_2_-IsoPs) as validated measures of oxidative status and the risk of weight gain [8] and type 2 diabetes [9]. Despite the conventional point of view that elevated F_2_-IsoP level reflects harmful oxidative stress [10, 11], we found that elevated F_2_-IsoP levels predicted lower risks of both weight gain [8] and type 2 diabetes [9]. Compared to Whites, African Americans have lower levels of urinary F_2_-IsoPs [8, 12] and this gap increases with obesity [12]. To explain these findings, we proposed a relationship between F_2_-IsoP levels and the intensity of mitochondrial oxidative metabolism. We reasoned that (a) F_2_-IsoPs are validated measures of the overall levels of reactive oxygen species (ROS) [13, 14] and (b) mitochondrial oxidative metabolism is the major endogenous source of ROS [15]. Thus, low F_2_-IsoP levels could reflect a metabolic profile that is known to be prevalent in African Americans, that is, slower mitochondrial oxidative metabolism [16–19]. We further hypothesized that systemic F_2_-IsoP levels present a race-specific metabolic phenotype that is linked to African ancestry. To test this hypothesis, we compared systemic F_2_-IsoP levels across three groups with a priori different proportion of African ancestry: non-Hispanic Whites (NHWs), US-born African Americans (AAs), and West African immigrants (WAI). African Americans are genetically related to the West African ancestry [20]; however, the percentage of African ancestry in self-identified AA is substantially lower (83%) as compared to WAI (95%) [21]. In this study, systemic F_2_-IsoP levels were measured in plasma. It has been established that both the urinary and the circulating levels of F_2_-IsoP present an accurate quantification of the endogenous production of these molecules [22]. We hypothesized that WAI, the group with the greatest proportion of African ancestry, will have the lowest systemic levels of F_2_-IsoPs among these three racial/ethnic groups, followed by AA.

## 2. Materials and Methods

### 2.1. Study Population

We used cross-sectional data from a previously conducted Study on Race, Stress, and Hypertension (SRSH). The study was designed to assess the differences in dietary, lifestyle, and psychosocial exposures in relation to blood pressure in a racially and ethnically diverse population. The methods of the study are described in detail elsewhere [23]. Briefly, the eligibility criteria included being an adult 25–74 years of age, self-identified as NHW, AA or WAI, and a permanent Georgia resident. For the WAI participants, an additional inclusion criterion was arrival to the US after the 18th birthday. Subjects were excluded if they did not give informed consent. There were 335 individuals who met the initial study inclusion criteria. Of those, 117 participants were excluded from the analyses due to missing values for plasma F_2_-IsoPs. All methods were reviewed and approved by the Institutional Review Boards of the Emory University and the Georgia State University.

### 2.2. Measurements of Plasma F_2_-IsoPs

All participants provided blood samples that were drawn into five 10 mL vacutainer tubes (2 sodium heparin tubes, 1 EDTA tube, and 2 red top tubes for serum collection) and immediately plunged into ice and protected from direct light. Plasma, serum, and buffy coat specimens were separated within 4–8 hours by centrifugation under refrigeration, aliquoted, frozen, and stored at −80°C. The aliquots were then shipped overnight on dry ice for molecular analysis by the Molecular Epidemiology and Biomarker Research Laboratory (MEBRL) at the University of Minnesota, Minneapolis, MN. Gas chromatography-mass spectrometry (GCMS) [24] was used to measure plasma free F_2_-IsoPs. The F_2_-IsoP were extracted from the plasma sample and deuterium (4)-labeled 8-iso-prostaglandin F_2_-alpha was used as an internal standard. The CV for the F2-isoprostane measurements was 9.7% for the “low” control (mean value = 47.48 pg/mL) and the CV for the “high” control is 11.2% (mean value = 89.87 pg/mL).

### 2.3. Other Measurements

Self-administered questionnaire collected data on demographic characteristics (age, sex, race/origin, and education), medical history (hypertension and use of medications), and lifestyle (physical activity and smoking) for all participants. The questionnaires were filled out and returned during the data collection sessions. Blood pressure and anthropometric measures (height and weight) were also taken during data collection sessions. The reported and measured BMI were highly correlated (*r* = 0.91). Hypertension was determined based on the blood pressure measurements (conducted by trained and certified staff using a standardized protocol) and self-report. Self-reported history of chronic diseases was broadly categorized into the following: diseases of heart (angina, congestive heart failure, myocardial infarction, hypertension, high blood cholesterol, pulmonary embolism, stroke, and thrombophlebitis), kidney (chronic kidney disease and kidney stone), endocrine diseases (diabetes, hypoglycemia and overactive thyroid), cancer (melanoma, skin cancer, and other cancers), and allergies (hay fever and atopic dermatitis). Medical history and hypertension were summarized as morbidity score, which represents a number of conditions reported by the participants and detected hypertension.

### 2.4. Statistical Analysis

The main objective of this analysis was to examine the relationship between race/ethnicity and circulating F_2_-IsoPs. To screen for potential confounders, study characteristics were compared between the three racial/ethnic groups using chi-square and Kruskal-Wallis tests for categorical and continuous variables, respectively (Table 1). Similarly, crude associations between study characteristics and plasma F_2_-IsoPs were examined using chi-square and Kruskal-Wallis tests for categorical and continuous variables, respectively (Table 2). Smoking was not considered as a potential confounder in this study, because only 5% of the study population were current smokers and smoking was shown not to be associated with F2-IsoP levels [23]. Linear regression models were used to examine the influence of potential confounders on the relationship between race/ethnicity and plasma F_2_-IsoPs (Table 3). Race/ethnicity was coded as a categorical variable (NHW/AA/WAI) with AA being the reference category. Model 1 included variables associated with both the exposure (race/ethnicity) and the outcome (plasma F_2_-IsoPs), except morbidity index. Model 2 includes significant predictors of plasma F_2_-IsoPs from Model 1 and morbidity index. Model 3 includes a modified morbidity index that incorporates obesity as a morbidity condition. This analysis was conducted using both the original scale variable for F_2_-isoprostanes and natural log-transformed variable. The comparison of F2-isoprostane distribution between the racial/ethnic groups stratified by comorbidity score was conducted using Kruskal-Wallis tests. All analyses were performed in SAS statistical software version 9.3 (SAS Institute Inc, Cary, NC).

## 3. Results

WAI were on average younger and had greater level of education (Table 1). Importantly, WAI were also healthier as compared to AAs and NHWs, reporting in their medical history fewer health conditions (Table 1). BMI did not vary among the racial/ethnic groups (*p* value 0.2). Plasma F_2_-IsoP levels were inversely associated with African ancestry gradient and were lowest among WAI and highest among NHW (Table 2). We followed the conventional rule for confounder screening by examining the associations of different study characteristics with both the main exposure (race/ethnicity, Table 1) and the outcome (F_2_-IsoP levels, Table 2). The data presented in Tables 1 and 2 indicate that age, BMI, and morbidity scores are potential confounders as these characteristics were associated with race/ethnicity and F_2_-IsoP levels. Obese individuals (BMI ≥ 30 kg/m^2^) on average had 35% greater levels of F_2_-IsoPs (*p* = 0.04) (Table 2). Among the reported chronic conditions, heart and kidney diseases as well as allergies were associated with greater F_2_-IsoP levels (*p* < 0.01), whereas endocrine conditions and cancer showed a trend toward positive association (*p* values 0.09 and 0.07, resp.). This analysis revealed two major determinants of plasma F_2_-IsoP levels in this study population: race/ethnicity and the overall comorbidity expressed as morbidity score.

**Figure 1:**
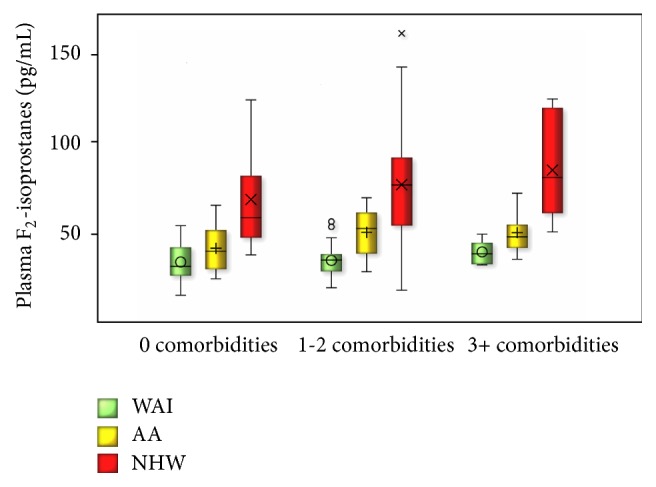
Distribution of plasma F_2_-isoprostanes in West African Immigrants (WAI), African Americans (AAs), and non-Hispanic Whites (NHWs) stratified by morbidity score. Boxplots are presented for each group, showing minimum, first quartile, median, third quartile, maximum, and the outliers (depicted as “o” and “x”). The *p* values for comparison of F2-isoprostane levels between racial/ethnic groups are < 0.05 within each strata of comorbidity score (Kruskal-Wallis tests).

The association between race/ethnicity and plasma F_2_-IsoP levels was examined by linear regression (Table 3). Age and sex were not significant predictors of F_2_-IsoP levels (Model 1, Table 3) and therefore were excluded from the final model. This exclusion did not substantially change the estimates of beta coefficients for race/ethnicity (data not shown). BMI was a significant predictor of F_2_-IsoP levels only in the absence of morbidity score (Models 1 and 2, Table 3). As BMI correlated with morbidity score (*r* = 0.53, *p* < 0.001), we included obesity into the morbidity score in the final model (Model 3, Table 3). Overall, WAI had the lowest levels of plasma F_2_-IsoP and AA had higher F_2_-IsoP levels as compared to WAI but lower than NHW (Table 3). When stratified by morbidity score that included obesity, F_2_-IsoP levels were lowest among WAI, followed by AA, and the highest levels were among NHW among healthy nonobese participants as well as among participants with comorbidities (Figure 1). Kruskal-Wallis showed significant differences across the groups within each comorbidity category (*p* values < 0.001).

## 4. Discussion

The main finding of this study is the inverse relationship between African ancestry gradient and systemic F_2_-IsoP levels. The observed association was statistically significant and in the hypothesized direction. This inverse relationship is confirmed by both the crude and regression analyses, after adjustment for potential confounders. Specifically, age, gender, BMI, and comorbidities were examined as potential confounders based on the previous studies showing associations between these variables and either race or systemic F_2_-IsoPs levels. The stratified analysis demonstrated that, among healthy nonobese participants, the gradient of African ancestry inversely correlates with plasma F_2_-IsoP levels. Stratification by obesity and comorbidity status may be important, because the differences in plasma F_2_-IsoP levels could be attributable to accumulated chronic conditions [10]. The observations that nonobese healthy individuals differ with respect to their plasma F_2_-IsoP levels and that the difference persists at greater comorbidity levels suggest that these differences reflect genetic variability rather than life style or health status. In essence, we propose that systemic F_2_-IsoP levels present a phenotypical characteristic that similarly to metabolism and obesity is influenced by genetic background but can be modified by lifestyle or pharmacological interventions. Below we discuss the biological meaning of systemic F_2_-IsoPs levels and a potential for intervention.

As systemic levels of F_2_-IsoPs are validated indices of oxidative status [13, 14], the greater levels of these compounds in circulation and in urine have been interpreted as indicators of harmful oxidative stress. This point of view draws on multiple findings of elevated systemic F_2_-IsoP levels in patients with different chronic conditions [10]. In this study, we also found that plasma F_2_-IsoP levels correlate with comorbidity index. However, these correlations may reflect the consequence rather than a cause of health problems, because prospective evidence does not support the associations between elevated F_2_-IsoP levels and onset of chronic diseases in three independent cohorts [8, 9, 25–27]. Moreover, physical activity, which is a known preventive factor for obesity and diabetes [28], leads to increased levels of systemic F_2_-IsoPs [29, 30]. Thus, there is an apparent contradiction between the conventional interpretation of systemic F_2_-IsoP levels as oxidative stress and epidemiological evidence from prospective studies and studies of physical activity portraying these biomarkers as a favorable phenotype.

We proposed that the molecular mechanisms underlying individual differences in systemic F_2_-IsoP levels as markers of ROS generation are human variability in oxidative metabolism [31], which is the major endogenous source of ROS [15]. Several of our previously published findings indicate that systemic F_2_-IsoP levels may specifically reflect the intensity of mitochondrial fatty acid oxidation [30, 32]. We demonstrated that urinary F_2_-IsoP levels are positively associated with the acylcarnitine species that are increased at the physiological state with enhanced fat oxidation [32] and that their basal level increases after aerobic training [30]. Taking into account that AA have lower levels of fat oxidation [18, 33] and mitochondrial metabolism [16–19], we propose that these phenotypes are associated with African ancestry and are reflected by low F_2_-IsoP levels. Other findings support the notion that genetic background might be connected to systemic F_2_-IsoP levels via intensified or slow metabolic phenotype. For example, children with sickle cell disease have hyperactive metabolism, which is associated with greater F_2_-IsoP levels [34, 35]. In contrast, patients with Down syndrome have lower metabolic rate and are prone to obesity [36], which corresponds to our findings of a trend of lower urinary F_2_-IsoP levels in Down syndrome patients [37]. On the other hand, in a relatively homogenous ethnic population of Danish monozygotic and dizygotic twins (with similar genetic background), the heritability of urinary F_2_-IsoP levels was low (~22%) [38]. The Danish study suggests that systemic F_2_-IsoP levels can be a modifiable factor, corroborating our hypothesis that systemic F_2_-IsoP levels are related to mitochondrial oxidative metabolism, a metabolic characteristic that is flexible and can be targeted by lifestyle and/or pharmacological interventions [39–41].

The potential for interventions in addressing low mitochondrial metabolism and muscle fat utilization has been demonstrated by small studies showing that these metabolic traits can be reversed. For example, a study by Cortright et al. [18] showed that lean AA women had lower levels of muscle fat oxidation as compared to White women and that exercise training had a stronger effect on fat oxidation in AA women than in White women, suggesting that fat oxidation could be an effective target for race-specific interventions. Another example is a study by Williams et al. [42] demonstrating that metformin, a pharmacological agent known to increase fat oxidation, produced a two times greater reduction in HbA1c in diabetic African American compared (*n* = 7429) to White patients (*n* = 8783). However, the obvious barrier in assessing interventions that target mitochondrial metabolism and fat oxidation is absence of noninvasive means to monitor these phenotypical characteristics. Currently, muscle mitochondrial metabolism is assessed through muscle biopsy, which is invasive and cannot be applied for practical reasons. Similarly, the assessment of fat utilization by indirect calorimetry is time- and labor-consuming and also not applicable to population-based interventions. In contrast, systemic F_2_-IsoP levels can serve as a simple, noninvasive means of assessing these metabolic characteristics over time and would be widely applicable in both the research and clinical setting.

The important caveat, however, is the interpretation of systemic F_2_-IsoPs in weight loss interventions. As this study demonstrated and it also has been previously found in other populations [10], systemic F_2_-IsoP levels are associated with BMI. Moreover, weight loss, a desirable outcome, is associated with decrease in F_2_-IsoP levels [43]. This is in parallel with a known phenomenon of metabolic adaptation also known as adaptive thermogenesis, that is, compensatory changes in energy expenditure and fat oxidation that favor fat mass homeostasis [44]. It has been demonstrated that weight gain or loss leads to corresponding changes in energy expenditure and fat oxidation [33]. It is also known that metabolic response to weight perturbations differs between the individuals and is influenced by genetic background [45]. Currently, such metabolic adaptation favoring homeostasis of fat mass is one of the central problems in maintenance of weight loss [46]. Based on the phenomenon of metabolic adaptation, greater F_2_-IsoP levels should be used as a favorable phenotype only in relation to body fat mass or BMI. Such F_2_-IsoP/BMI index presents a noninvasive measure and may provide the basis for targeted interventions to prevent obesity and type 2 diabetes among populations of African descent.

## Figures and Tables

**Table 1:** Characteristics of the study population.

	WAI^a^ (*n* = 79)	AAs^a^ (*n* = 56)	NHWs^a^ (*n* = 83)	*p* value^b^
*Variable*				
Mean age (SD), years	42.5 (10.9)	49.1 (11.8)	48.2 (14.0)	0.002
Sex, % males	37.8	51.9	33.7	0.10
Education, % college graduates	52.0	32.1	43.9	0.04
BMI (SD), kg/m^2^	28.7 (6.7)	31.0 (6.7)	30.1 (6.5)	0.21
% Obese	39.7	50.0	42.2	0.50

*Medical history (Yes *%)				
Heart diseases	21.3	57.5	52.5	<0.001
Kidney diseases	0.0	9.1	15.3	0.001
Endocrine diseases	5.4	31.3	10.2	<0.001
Cancer	1.4	0.0	8.8	0.1138
Allergy	9.6	33.3	38.6	0.003

*Morbidity score, *%				
0 (number of categories reported)	74.0	28.3	21.1	<0.001
1	16.4	34.8	42.1	
2	8.2	15.6	31.6	
≥3	1.4	21.9	5.3	

^a^WAI: West African immigrants, AAs: African Americans, and NHWs: non-Hispanic Whites.

^b^ *p* values are presented for Chi-square test (categorical variables) and for Kruskal-Wallis rank sum test (continuous variables).

**Table 2:** Relationships between plasma F_2_-isoprostanes and study variables.

Continuous/ordinal variables	Spearman correlation coefficient	*p* value
Age	0.148	0.03
BMI	0.189	<0.01
Morbidity score^a^	0.437	<0.01

Categorical variables (Kruskal-Wallis test)	F_2_-isoprostane level mean (SD) (pg/mL)	*p* value

Race		
NHW	80.1 (34.9)	<0.001
AA	51.1 (20.1)	

NHW	80.1 (34.9)	<0.001
WAI	33.8 (9.0)	

AA	51.1 (20.1)	<0.001
WAI	33.8 (9.0)	

Sex		
Females	59.4 (35.8)	0.43
Males	51.1 (23.2)	

Education		
College graduate	50.5 (24.8)	0.26
Some college	59.2 (35.2)	
HS grads	60.9 (30.1)	
Less than HS	53.8 (31.0)	

Obesity		
Nonobese	51.1 (23.2)	0.04
Obese	69.2 (35.8)	

Heart disease		
Yes	58.6 (30.1)	<0.01
No	47.0 (24.9)	

Endocrine disease		
Yes	56.6 (23.9)	0.09
No	50.8 (28.3)	

Kidney disease		
Yes	82.6 (32.3)	<0.01
No	49.3 (25.9)	

Allergy conditions		
Yes	67.0 (28.4)	<0.01
No	46.4 (25.6)	

Cancer		
Yes	79.7 (45.0)	0.07
No	50.3 (26.4)	

^a^Morbidity score summarizes the number of comorbidities.

**Table 3:** Association between plasma F_2_-isoprostanes and African ancestry gradient.

	Beta coefficient (SE)					
	*p* value					
	Model 1		Model 2		Model 3^b^	
	A^a^	B^a^	A	B	A	B
AA (versus NHW)	−29.1 (4.3)	−0.436 (0.066)	−30.9 (4.4)	−0.468 (0.076)	−30.3 (4.4)	−0.456 (0.076)
	<0.001	<0.001	<0.001	<0.0001	<0.001	<0.0001

AA (versus WAI)	15.8 (4.5)	0.349 (0.069)	9.8 (4.5)	0.263 (0.077)	9.8 (4.5)	−0.265 (0.077)
	0.001	<0.001	0.03	0.0008	0.03	0.0007

Age (years)	−0.06 (0.1)	−0.001 (0.002)				
	0.65	0.69				

Sex (males versus females)	−5.3 (3.5)	−0.048 (0.054)				
	0.13	0.37				

BMI (kg/m^2^)	1.02 (0.3)	0.016 (0.004)	0.2 (0.3)	0.006 (0.005)	Excluded	Excluded
	<0.001	0.0001	0.40	0.22		

Morbidity score			3.15 (1.7)	0.051 (0.027)	3.9 (1.5)^b^	0.070 (0.026)^b^
			0.07	0.06	0.01	0.007

^a^F_2_-isoprostane variable presents the measurements on the original scale in A and on the natural log-transformed scale in B.

^b^In Model 3, obesity (BMI ≥ 30) was included in morbidity index.
